# Draft Genome Sequence of *Mycobacterium abscessus* subsp. *bolletii* INCQS 00594

**DOI:** 10.1128/genomeA.00896-13

**Published:** 2013-11-07

**Authors:** Sylvia Cardoso Leão, Cristianne Kayoko Matsumoto, Cristina Viana-Niero, Rommel Thiago Jucá Ramos, Adriana Ribeiro Carneiro, Maria Silvanira Barbosa, Karla Valéria Batista Lima, Maria Luiza Lopes, Vasco Azevedo, Artur Silva

**Affiliations:** Departamento de Microbiologia, Imunologia e Parasitologia, Escola Paulista de Medicina, Universidade Federal de São Paulo, São Paulo, São Paulo, Brazila; Instituto de Ciências Biológicas, Universidade Federal do Pará, Belém, Pará, Brazilb; Seção de Bacteriologia e Micologia, Instituto Evandro Chagas, Ananindeua, Pará, Brazilc; Instituto de Ciências Biológicas, Universidade Federal de Minas Gerais, Belo Horizonte, Minas Gerais, Brazild

## Abstract

An epidemic of surgical-site infections by a single strain of *Mycobacterium abscessus* subsp. *bolletii* affected >1,700 patients in Brazil from 2004 to 2008. The genome of the epidemic prototype strain *M. abscessus* subsp. *bolletii* INCQS 00594, deposited in the collection of the National Institute for Health Quality Control (INCQS), was sequenced.

## GENOME ANNOUNCEMENT

*Mycobacterium abscessus* is a rapidly growing mycobacterium and an emerging human pathogen causing pulmonary infections, especially in patients with cystic fibrosis and other underlying lung disorders (1). *M. abscessus* is also a major cause of sporadic and clustered outbreaks of community-acquired and health care-associated skin and soft tissue infections (2). Recently, two subspecies were recognized within *M. abscessus*: *M. abscessus* subsp. *abscessus* and *M. abscessus* subsp. *bolletii* (3). The latter subspecies includes strains of two previously described taxa, *M. massiliense* and *M. bolletii* (4–6).

A prolonged nationwide epidemic of surgical infections related to video surgeries occurred in Brazil between 2004 and 2008 (7). The analysis of isolates obtained in different states confirmed that the epidemic was caused by a single strain of *M. abscessus* subsp. *bolletii* (8–12). One isolate, obtained from a patient who underwent laparoscopic surgery in 2004 in Belém, Pará (PA), was deposited as the epidemic prototype strain in the collection of the National Institute for Health Quality Control (INCQS) under the name INCQS 00594 (8). This isolate was shown to carry a circular plasmid of the IncP-1β group, named pMAB01, which was the subject of a previous publication by Leão et al. (13).

The genome of *M. abscessus* subsp. *bolletii* INCQS 00594 was sequenced from a fragment library using the 454 GS FLX sequencer (Roche, Nutley, NJ) and the GS FLX Titanium sequencing kit XL+ at the Centre for Advanced Technologies in Genomics (CATG), Chemistry Institute, São Paulo University. A total of 779,074 reads (558,522,880 bp) were extracted from the .sff file with the script “sff_extract_0_2_13” (http://bioinf.comav.upv.es/sff_extract/), which represents a genomic coverage of ~111×. The reads were assembled using the MIRA software (14), generating 102 contigs with an N_50_ of 664 kb. Eighteen contigs matched sequences in the plasmid pMAB01 (GenBank accession no. CP003376), and these were not considered here. The remaining sequences were annotated via RAST (15). The draft genome sequence of *M. abscessus* subsp. *bolletii* INCQS 00594 has 4,885,146 bp, an overall G+C content of 64.24%, 4,864 open reading frames (ORF), and one rRNA operon.

### Nucleotide sequence accession numbers.

This genome project has been deposited in DDBJ/EMBL/GenBank under the accession no. AUVF00000000. The version described in this paper is the first version, AUVF01000000.
